# Young adult male carriers of the fragile X premutation exhibit genetically modulated impairments in visuospatial tasks controlled for psychomotor speed

**DOI:** 10.1186/1866-1955-4-26

**Published:** 2012-11-13

**Authors:** Ling M Wong, Naomi J Goodrich-Hunsaker, Yingratana McLennan, Flora Tassone, Danielle Harvey, Susan M Rivera, Tony J Simon

**Affiliations:** 1 MIND Institute and Department of Psychiatry and Behavioral Sciences, University of California, Davis, 2825 50th Street, Sacramento, CA, 95817, USA; 2 MIND Institute and Department of Biochemistry and Molecular Medicine, University of California, Davis, 2825 50th Street, Sacramento, CA, 95817, USA; 3 Division of Biostatistics, Department of Public Health Sciences, School of Medicine, One Shields Avenue, Med Sci 1-C, University of California, Davis, CA, 95616, USA; 4 Center for Mind and Brain, 267 Cousteau Place, University of California, Davis, CA, 95618, USA

**Keywords:** X-linked genetic disease, Psychomotor performance, FMR1

## Abstract

**Background:**

A previous study reported enhanced psychomotor speed, and subtle but significant cognitive impairments, modulated by age and by mutations in the fragile X mental retardation 1 (FMR1) gene in adult female fragile X premutation carriers (fXPCs). Because male carriers, unlike females, do not have a second, unaffected FMR1 allele, male fXPCs should exhibit similar, if not worse, impairments. Understanding male fXPCs is of particular significance because of their increased risk of developing fragile X-associated tremor/ataxia syndrome (FXTAS).

**Methods:**

Male fXPCs (n = 18) and healthy control (HC) adults (n = 26) aged less than 45 years performed two psychomotor speed tasks (manual and oral) and two visuospatial tasks (magnitude comparison and enumeration). In the magnitude comparison task, participants were asked to compare and judge which of two bars was larger. In the enumeration task, participants were shown between one and eight green bars in the center of the screen, and asked to state the total number displayed. Enumeration typically proceeds in one of two modes: subitizing, a fast and accurate process that works only with a small set of items, and counting, which requires accurate serial-object detection and individuation during visual search. We examined the associations between the performance on all tasks and the age, full-scale intelligent quotient, and CGG repeat length of participants.

**Results:**

We found that in the magnitude comparison and enumeration tasks, male fXPCs exhibited slower reaction times relative to HCs, even after controlling for simple reaction time.

**Conclusions:**

Our results indicate that male fXPCs as a group show impairments (slower reaction times) in numerical visuospatial tasks, which are consistent with previous findings. This adds to a growing body of literature characterizing the phenotype in fXPCs who are asymptomatic for FXTAS. Future longitudinal studies are needed to determine how these impairments relate to risk of developing FXTAS.

## Background

Fragile X syndrome (FXS) is the most common hereditary cause of intellectual disability in males, and is the leading single genetic cause of autism. The disorder is caused by methylation of an expanded trinucleotide CGG (>200) repeat in the promoter of the fragile X mental retardation 1 (FMR1) gene, which is located on the q27.3 site of the X chromosome [1], and is associated with low or absent levels of FMR1 mRNA and protein (FMRP). FMRP binds to as much as 4% of all mRNA in mammalian brains [2], and is thought to play a crucial role in synapse development and plasticity. Indicative of the importance of FMRP, the level of cognitive ability correlates with the level of FMRP in males with FXS [3]. Carriers of the fragile X premutation (fXPCs) are defined by the presence of 55 to 200 CGG repeats within the FMR1 gene, which results in a three- to eight-fold increase in FMR1 mRNA levels in leukocytes [4], but little or no reduction in FMRP. It is estimated that 1 in 260–813 males and 1 in 113–259 females carry the premutation allele [5], and approximately 40% of male fXPCs and 8-16% of female fXPCs develop fragile X-associated tremor ataxia syndrome (FXTAS) [6]. FXTAS is a late-onset (>50 years old) neurodegenerative disorder associated with tremors, gait ataxia, parkinsonism, and impairments in short-term memory and executive functions [7]. It is thought to result from RNA toxicity due to elevated FMR1 mRNA levels [8].

Recent indications that the fragile X premutation allele negatively affects neurodevelopment during an individual’s life span shifted the research field away from evaluating the premutation solely as a risk factor for neurodegeneration (that is, FXTAS), and towards elucidating the consequences of this altered neurodevelopment. Some studies suggest that fXPCs younger than 50 years of age are largely cognitively unaffected by the mutation [9-12]; however, adult female fXPCs tend to have faster oral and manual motor psychomotor speed than adults not carrying the premutation [13,14]. This suggests that a lack of difference in performance between female fXPCs and controls in cognitively demanding, non-standardized tasks may actually represent slowing of cognitively modulated performance times, which is masked by enhanced simple reaction time.

Studies reporting no differences between male fXPCs and controls have used tasks such as the Controlled Oral Word Generation Task, Stroop Color-Word Task, sections of the Behavioral Dyscontrol Scale, and certain selective attention tasks [12,15-20], and some of these controlled for manual motor performance using independent assessment by the Purdue Pegboard Test or CATSYS system (http://www.catsys.dk/purchase.htm) as covariates [15-17]. Because male fXPCs are at increased risk for developing FXTAS [6], which is characterized by motor impairment, any reaction time differences between fXPCs and controls could be due to cognitive slowing or motor slowing. Thus, it is particularly important to control for psychomotor speed of male fXPCs when assessing performance on cognitive tasks.

Because fXPCs are at increased risk of developing FXTAS, which is a neurodegenerative disorder often accompanied by attentional control impairments, it may be prudent to determine whether similar cognitive impairments are observable in fXPCs asymptomatic for FXTAS. If cognitive impairments precede motor impairments, cognitive impairments might be used as a biomarker for risk of disease progression. To understand the cognitive phenotype of fXPCs, we may look to the similarities between fXPCs and individuals with FXSc, because one way to view the effects of all FMR1 mutations is to view them as existing on a phenotypic spectrum that is modulated by FMR1 dosage in terms of CGG repeats and gender. Specifically, FMR1 dosage increases with CGG repeat length, and males have a higher FMR1 ‘dose’ than females, because the premutation allele is expressed in all of their cells, and they lack a second, unaffected FMR1 allele. In support of a genetically modulated phenotypic spectrum are the findings that individuals with FXS or the premutation (with or without FXTAS) share symptoms of executive function impairments [21-23] that are modulated by CGG repeat length [18,19,24-26], FMR1 mRNA [27], and FMRP [28-32]. Additionally, CGG repeat length relates to age of onset of FXTAS [33] and to degree of brain atrophy [34].

Individuals with FXS exhibit difficulties in understanding space, time, and numbers_,_ which result in characteristic quantitative and numerical impairments [35-37]. Functional brain activation during arithmetic processing in females with FXS was found to be related to FMRP expression, suggesting that decreased FMRP expression underlies impairments in mathematics performance in individuals with FXS [38]. Similarly, adult female fXPCs also have arithmetic impairments [39], and impairments in judging relative magnitude and enumeration that are modulated by CGG and age [40,41]. Positron emission tomography imaging in adult female fXPCs indicates hypometabolism of the right parietal, temporal, and occipital association areas [42], suggesting that these impairments may be due to abnormal functioning of brain regions involved in visuospatial attention. Additionally, girls with FXS have impaired performance on ‘where’ tasks [37], and men with FXS and adult fXPCs have specific magnocellular (M) pathway impairment [43-45]. CGG knock-in mice, a model for fXPCs, also show similar spatiotemporal processing impairments [46-48]. Thus, although individuals with the full mutation or premutation are thought to be affected via different mechanisms (FMRP deficiency and RNA toxicity, respectively), evidence suggests that they share a common impairment of spatial and temporal processing that affects higher-level processing (for example, numerical thinking and arithmetic).

The purpose of this study was to determine whether young adult male fXPCs, asymptomatic for FXTAS, exhibit impairments in numerical visuospatial tasks. We used two psychomotor speed tasks (manual and oral), which allowed us to control for baseline differences in response time. We also used two visuospatial tasks: magnitude comparison and enumeration. These tasks allowed us to examine judgments of relative magnitude (numerical distance effect) and the ability to indicate the number of presented items. Our previous work with adult female fXPCs in the same age range reported impairments that were modulated by CGG repeat length and age, indicating that these tasks are sensitive to FMR1 allele variants [40,41]. Because females have a second, unaffected FMR1 allele that is expressed randomly in 50% of their cells, they should be less affected than males. Thus, we hypothesized that male fXPCs would be impaired relative to HCs and would be more cognitively affected than female fXPCs.

## Methods

### Ethics approval

This study was approved by the institutional review board, and conformed to institutional and federal guidelines for the protection of human participants. Written informed consent was obtained before participation from all participants.

### Recruitment

Participants were recruited through the NeuroTherapeutics Research Institute (NTRI) at the Medical Investigation of Neurodevelopmental Disorders (MIND) Institute at the University of California, Davis Medical Center, and from the community through recruitment advertisements. All participants had normal, or corrected to normal, vision.

Exclusion criteria were: acute medical condition such as renal, liver, or cardiac or other disease that may be associated with brain atrophy or dysfunction, current or past history of major DSM-IV Axis I psychiatric disorder, history of head trauma, toxic encephalopathy, encephalitis, or bacterial meningitis, history of alcoholism or drug problem, and use of current medication that might affect cerebral blood flow (for example, beta blockers).

In total 44 males aged 19 to 45 years (26 healthy controls (HCs) and 18 fXPCs) were recruited. The mean ± SD age was 31.0 ± 6.94 years for HCs and 30.72 ± 6.51 years for fXPCs (Table 1). The two groups did not differ in age (t = 0.18, P = 0.86), full-scale IQ (FSIQ) (t = 0.37, P = 0.71), verbal IQ (t = 0.58, P = 0.57), or performance IQ (t = 0.09, P = 0.93).

**Table 1:** Participant descriptive statistics and FMR1 measures

	**Healthy controls**			**fXPCs**			**t**	**P-value**
	**Mean ± SD**	**Range**	**n**	**Mean ± SD**	**Range**	**n**		
Age	31.10 ± 6.94	19 to 41	26	30.72 ± 6.51	20 to 45	18	−0.18	0.86
FSIQ	117.50 ± 18.65	85 to 148	16	115.29 ± 12.98	94 to 136	14	−0.37	0.71
CGG repeats	29.09 ± 3.87	20 to 40	22	88.28 ± 16.21	55 to 118	18	15.12	<0.001

FSIQ, full-scale intelligence quotient; fXPC, fragile X premutation carrier.

### Psychological assessment

FSIQ was measured using either the Wechsler Adult Intelligence Scale, third edition (WASI-III) [49] or the Wechsler Abbreviated Scale of Intelligence (WASI) [50]. FSIQ data were not collected from all participants because of timing constraints, hence data were available for 16 of the 26 HCs and 14 of the 18 fXPCs.

### Molecular analysis

Genomic DNA was isolated from peripheral blood leukocytes using standard methods (Puregene Kit; Gentra Inc., Valencia, CA, USA). Repeat length was determined using Southern blot analysis and PCR amplification of genomic DNA as described previously [51].

### Behavioral tasks

All tasks were presented on a computer (2 GHz Intel Core 2 Duo HP Compaq dc7700 Small Form Factor PC) equipped with 1 GB of RAM and running SuperLab software (version 4.0.7b; Cedrus Corporation, San Pedro, CA, USA).

#### Simple reaction time

The stimuli included a drawing of a house that was 190 mm in height and 79 mm wide, with an entrance that was 54 mm high and 32 mm wide on the monitor, which was placed 600 mm from the participant. For each trial, participants were asked to indicate as quickly as possible, by pressing a single button for the manual motor version of the task or by speaking the word ‘Go’ into a microphone for the oral motor version of the task, whenever a picture of a friendly alien appeared at the right or left side of the entrance. For the manual motor version, participants used their dominant hand. The alien figure was 54 mm tall and 18 mm in the widest extent. This image remained on the screen until the participant responded. The version order was randomized across participants. Each version of the task consisted of 60 consecutive trials. Delays between trials were set to one of three intervals (400, 800, or 1200 ms), which were presented in random order to minimize anticipatory responses. Response time was recorded as the primary dependent variable.

#### Magnitude comparison (distance effect)

Participants were asked to indicate which of two blue bars was the larger. To begin each trial, the participant looked at the fixation point on the computer monitor. Once the participant was ready, the stimuli were presented. The bars were vertically oriented, horizontally offset from fixation by 30 mm, and centered at the level of fixation. Each bar was 20 mm wide, and varied in height from 10 to 120 mm in 10 mm increments. Participants pressed one of two buttons as quickly and accurately as possible to indicate which stimulus was larger. Stimuli were presented until the participant responded or until 7 seconds had elapsed. Response time and error rate were recorded as the dependent variables.

The total number of trials was 120, divided evenly into two blocks of 60 trials. There was a short rest period between the two blocks of trials. The 60 trials consisted of 10 trials at each of the 6 possible differences between the heights of the 2 bars (10, 20, 30, 50, 60, and 70 mm). Participants were seated 600 mm from the screen; thus the possible size differences between the bars were 0.95, 1.91, 2.86, 4.76, 5.71, and 6.65 degrees of visual angle. To reduce the overall number of trials, no height difference of 40 mm was included in the experiment. We reasoned that because height differences of 40 mm were in the center of our range, they would not contribute much to our understanding of how small and large height differences affected visuospatial cognition. These were the precise pairs used to represent the following distances: 10 mm (10 to 20, 20 to 10, 20 to 30, 30 to 20, 60 to 70, 70 to 60, 70 to 80, 80 to 70, 90 to 100, 100 to 90 mm); 20 mm (10 to 30, 20 to 40, 30 to 10, 40 to 20, 50 to 70, 60 to 80, 70 to 50, 80 to 60, 90 to 110, 110 to 90 mm); 30 mm (10 to 40, 30 to 60, 40 to 10, 40 to 70, 50 to 80, 60 to 30, 70 to 40, 80 to 50, 80 to 110, 110 to 80 mm); 50 mm (10 to 60, 20 to 70, 30 to 80, 40 to 90, 50 to 100, 60 to 10, 70 to 20, 80 to 30, 90 to 40, 100 to 50 mm); 60 mm (10 to 70, 20 to 80, 30 to 90, 40 to 100, 50 to 110, 70 to 10, 80 to 20, 90 to 30, 100 to 40, 110 to 50 mm); and 70 mm (10 to 80, 20 to 90, 30 to 100, 40 to 110, 50 to 120, 80 to 10, 90 to 20, 100 to 30, 110 to 40, 120 to 50 mm). These pairs were each presented once during each block. The presentation of the six height differences were counterbalanced within groups of six trials, so that the possible height differences were evenly distributed across the 60 trials pseudorandomly.

#### Enumeration

Participants were asked to say into a microphone as quickly and accurately as possible the number of objects seen on the screen. To begin each trial, the participant looked at the fixation point on the computer monitor. Once the participant was ready, the stimuli were presented. Target stimuli consisted of one to eight bright green rectangles, measuring 0.25 × 0.24 degrees on a red background square with 2-degree sides when viewed from a distance of 600 mm. Targets were visible on the screen until the participant responded, at which point the vocal response terminated the trial and the timer. The experimenter, who was seated in a position from which the screen was not visible, recorded the participant’s response using the computer’s keyboard. Response time and error rate were recorded as the dependent variables.

For each numerosity (1 to 8), there were 20 different stimuli in which the requisite number of targets was placed randomly within an invisible 4 × 4 grid. The experiment consisted of 5 blocks of 16 trials. All possible numerosities (1 to 8) were randomly distributed within a block for a total of 80 trials. A rest period was provided after every block.

### Statistical analysis

For all analyses, degrees of freedom were adjusted using the Welch procedure for one-way analysis of variance (ANOVA) when the equality of variance assumption was violated. For repeated-measures ANOVA, Greenhouse–Geisser corrections were used to correct for violations of the sphericity assumption. Median reaction times were used when plotting the data, but the reaction times were log-transformed so the analyses better met the assumptions of the model. Analyses were conducted using SPSS software (SPSS, Chicago, IL, USA) and a P<0.05 was considered significant.

#### Simple reaction time

Both manual or oral motor reaction time were measured. Within each version, trials with reaction times greater than 3 times the interquartile range (IQR), less than 3 times the IQR, or less than or equal to 150 ms (anticipatory responses) were excluded from the analyses. Results were calculated as the median of the reaction times across each condition delay. Repeated-measures ANOVA with delay (400, 800, 1200 ms) as the within-participant factor, and group (HC and fXPC) as the between-participant factor, were performed. Correlations between the median simple reaction time across all trials and age (for both groups) and CGG repeat length (in the male fXPCs) were computed within groups.

#### Magnitude comparison (distance effect)

Data from the distance effect task measured magnitude comparison as assessed by response time (in ms) and error rate. These data were blocked in accordance with the six possible height differences of 10, 20, 30, 50, 60, and 70 mm. As in our previous studies [52], anticipatory responses and outliers were excluded from the analyses. Anticipatory responses were determined to be any response equal to or less than 150 ms. Outliers were determined to be a response greater than 3 times the IQR or less than 3 times the IQR of the response times at a specific height difference (for example, 10, 20, 30, 50, 60, or 70 mm). After excluding trials with outlier responses, the median reaction time was calculated for each trial condition. To parse basic psychomotor speed from cognitive load, the distance effect reaction time was adjusted by dividing the median reaction time for each height difference by the median reaction time from the manual motor simple reaction time task.

One-way ANOVA and repeated-measures ANOVA were used to assess differences between the groups. To determine the range of reliable ‘distance effect’, repeated-measures ANOVA models were used for the data within a group in a sequential manner, starting with distances from 10 to 30 mm and adding the next largest distance until a quadratic trend for distance was identified [52-54]. Once the ‘distance effect’ was identified, the analyses focused on those distances. For each individual participant, a simple linear-regression model with adjusted reaction time as the outcome and distance as the independent variable was used to get an estimate of the participant-specific intercept (estimated intercept). These values for each participant correspond to the estimated adjusted reaction time at a distance of 0 mm. Using one-way ANOVA, these values were then compared between the groups. Correlations between outcomes and age (for both groups) and CGG repeat length (in the male fXPCs) were computed within groups.

#### Enumeration

Data from the enumeration task measured visuospatial processes as assessed by response time and error rate. These data were blocked according to the numerosity (1–8). As in our previous studies [52], anticipatory responses and outliers were excluded from the analyses. Anticipatory responses were determined to be any response time less than or equal to 150 ms. Outliers were determined as a response time greater than 3 times the IQR or less than 3 times the IQR of the response times at each numerosity. After excluding trials with outlier responses, the median reaction time was calculated for each trial condition. To parse basic psychomotor speed from cognitive load, this enumeration reaction time was adjusted by dividing the median reaction time for each numerosity by the median reaction time from the oral motor simple reaction time task.

One-way ANOVA and repeated-measures ANOVA were used to assess differences between the groups on the two tasks. To estimate subitizing ranges for each group, repeated-measures ANOVA models were used for the data within a group in a sequential manner, starting with 1 to 3 items and adding the next largest number until a quadratic trend for the number of items was identified. Once the subitizing range was identified, the remaining range was identified as the counting range, and analyses focused on both ranges. Simple linear-regression models, with adjusted reaction times as the outcome and number of items as the independent variable, were used for each participant’s data to get estimates of participant-specific slopes during the subitizing and counting ranges. These values corresponded to how quickly the reaction times increased as the number of items increased. These values were then compared between the groups using one-way ANOVA. Correlations between outcomes and age (for both groups) and CGG repeat length (in the male fXPCs) were computed within groups.

## Results

### Molecular analyses

Molecular data were available for 22 of 26 HCs and for all 18 fXPCs. At the onset of this study, participants were given the option of supplying a blood or saliva sample for genetic analyses. Once it became apparent that saliva samples were insufficient to determine CGG repeat length conclusively, all participants were required to supply blood samples. Thus, molecular data were not available from the four initial control participants. There were no differences between groups in age or FSIQ score, and as expected, fXPCs had larger CGG repeat lengths than HCs (Table 1).

### Simple reaction time

**Figure 1:**
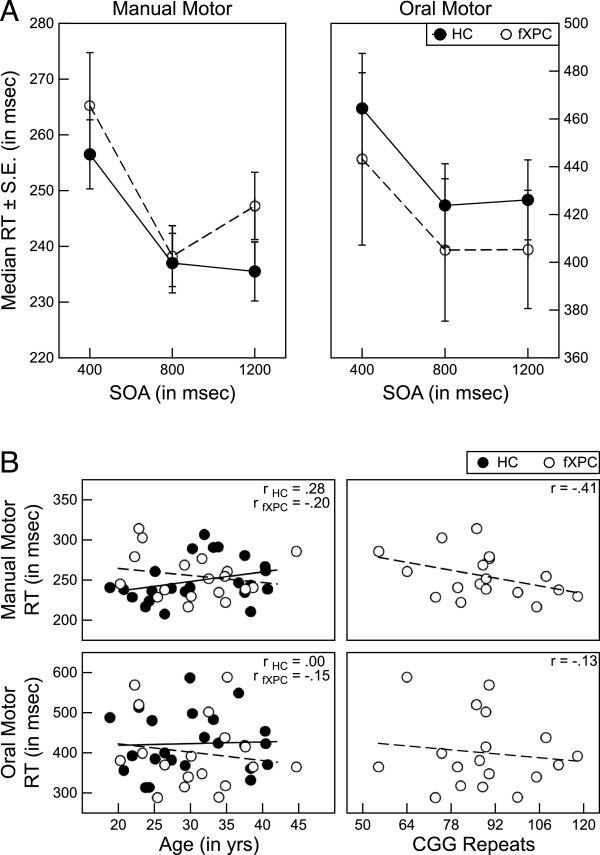
**Analyses of simple reaction time for male fragile X premutation carriers (fXPCs) and healthy controls (HCs).** (**A**) Group analyses of response time showed similar responses for fXPCs and HCs on the manual (P = 0.37) and oral (P = 0.38) motor reaction time tasks. (**B**) Assessment of the association between performance and age (both groups) and CGG repeat length (in the male fXPCs); no significant association was seen.

The simple reaction time tasks assessed the ability of each participant to make rapid responses with minimal cognitive demands needed for accurate performance. Responses from one male HC on the manual motor version and from a different male HC on the oral motor version were identified as outliers, so they were removed from the analyses. In the remaining participants, the number of excluded trials (mean ± SD) in the manual version was 2.08 ± 1.19 for HCs and 1.17 ± 0.86 for fXPCs, which differed significantly (t = 2.92, P = 0.005), and in the oral version it was 2.84 ± 2.21 for HCs and 3.11 ± 2.52 for fXPCs, which did not differ significantly (t = 0.36, P = 0.72). A repeated-measures ANOVA showed that male fXPCs responded similarly to the HCs on the manual (F_(1,41)_ = 0.81, P = 0.37) and oral (F_(1,41)_ = 0.80, P = 0.38) motor reaction time task (Figure 1A). Overall median reaction times across all trials for each task version within each group were used to assess the association between performance and age (both groups) and CGG repeat length (in the male fXPCs), and there were no significant associations (Figure 1B). The correlation matrix is presented in Table 2.

**Table 2:** **Correlation matrix**^**a**^

**Variable**	**HC**	**fXPC**	
	**Age**	**Age**	**CGG**
Age	–	–	–
CGG repeat length	NA	−0.29	–
Manual motor	0.28	−0.21	−0.41
Oral motor	0.00	−0.15	−0.13
Magnitude comparison	0.39	0.07	0.30
Subitizing	−0.08	−0.03	−0.02
Counting	0.25	0.02	−0.23

HC, healthy control; fXPC, fragile X premutation carrier; NA, not available.

^a^Correlation is significant at the 0.05 level (one-tailed).

### Magnitude comparison (distance effect)

**Figure 2:**
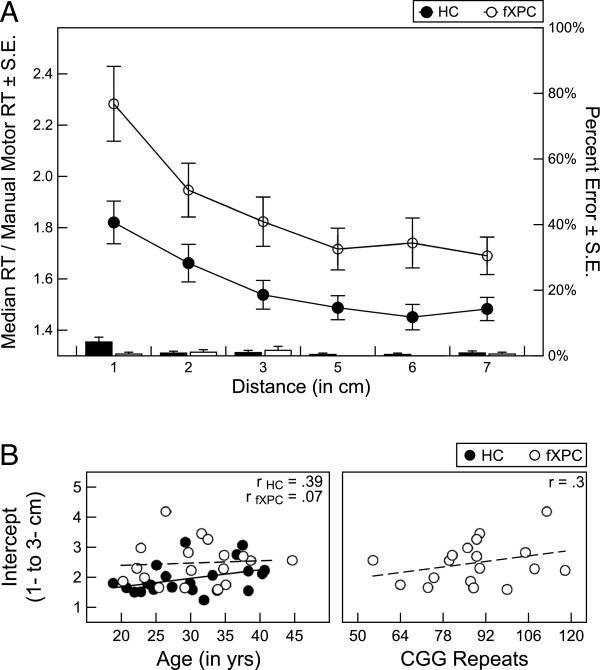
**Analyses of magnitude comparison response times for fragile X premutation carriers (fXPCs) and healthy controls (HCs).** (**A**) Group analyses of response time, controlled for manual motor simple reaction time performance, showed that male fXPCs, as a group, responded more slowly than HCs (P = 0.008). Response times adjusted for simple reaction time increased as the difference between the two blocks decreased (P<0.001), which did not differ between the two groups (P = 0.10). (**B**) Assessment of the association between performance and age (both groups) and CGG repeat length (in the male fXPCs); no significant associations were seen.

Responses from two male HCs were identified as outliers, so they were removed from the analyses. For the remaining participants, the mean ± SD number of excluded trials was 1.67 ± 1.52 for HCs and 0.67 ± 0.91 for fXPCs, which differed significantly (t = 2.65, P = 0.01). There was no difference in error rates between the two groups (F_(1,40)_ = 1.75, P = 0.19). The mean error rate ranged from 0.88% ± 3.0% at 70 mm to 4.21% ± 7.22% at 10 mm for HCs and from 0.56% ± 2.4% to 0.56% ± 2.4%, respectively, for male fXPCs. Using a repeated-measures ANOVA, we found that there was a significant difference in reaction times between the two groups (F_(1,40)_ = 8.09, P = 0.008; Figure 2A); reaction times increased as the difference between the two blocks decreased (F_(5,200)_ = 30.07, P<0.001), and did not differ between the groups (F_(5,200)_ = 1.86, P = 0.10). This indicates that reaction times slowed as the difference between the two blocks decreased, and both groups slowed at the same rate. In three separate analyses, the main effects of group and distance remained significant (P<0.05), and the interaction between group and distance on reaction time remained insignificant, when 1) no correction for simple reaction time was applied, 2) outlier trials were not excluded, and 3) no correction for simple reaction time was applied and outlier trials were not excluded (results not shown).

Analyses to identify a reliable ‘distance effect’ were performed in each group separately. For the HCs, a significant quadratic trend emerged for 10 to 50 mm, indicating a ‘distance effect’ range from 10 to 30 mm (F_(1,23)_ = 7.34, P = 0.01). Similarly, for the male fXPCs, a significant quadratic trend emerged for 10 to 50 mm, indicating a ‘distance effect’ range from 10 to 30 mm (F_(1,17)_ = 15.86, P<0.001). Because quadratic trends were identified over the range of 10 to 50 mm for both groups, we focused on distances from 10 to 30 mm, where the changes in reaction times were linear, for the remaining comparisons. Intercepts for the linear fit lines through the points at distances of 10 and 30 mm were estimated for each person.

To parse basic psychomotor speed from cognitive load, the median reaction time for each distance was divided by the median reaction time from the manual motor simple reaction time task, thus the intercept is expressed in terms of an arbitrary unit (AU). The intercept (mean ± SD) was 2.00 ± 0.49 AU for HCs and 2.48 ± 0.70 AU for male fXPCs. Intercepts were significantly worse for male fXPCs than for HCs (t = 2.83, P<0.01). We also calculated the intercepts using raw median reaction times to confirm these results. The mean intercept was 493.01 ± 121.85 ms for HCs and 632.54 ± 191.44 ms for male fXPCs, which were significantly different (t = 2.88, P<0.01).

Further investigation into the intercepts within each group assessed the association between these measures and age (both groups) and CGG repeat length (in the male fXPCs). There were no significant associations (Figure 2B). The correlation matrix is presented in Table 2.

### Enumeration task

**Figure 3:**
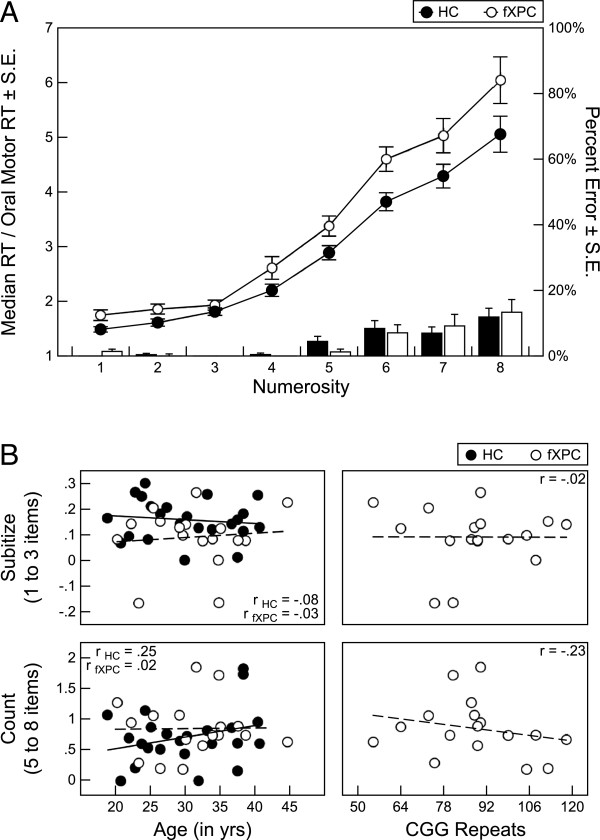
**Analyses of enumeration response time for fragile X premutation carriers (fXPCs) and healthy controls (HCs).** (**A**) Group analyses of response time, controlled for oral motor simple reaction time performance, showed that male fXPCs, as a group, responded more slowly than HCs (P = 0.02). Response times adjusted for simple reaction time increased as the number of items to enumerate increased (P<0.001), which did not differ between the two groups (P = 0.60). (**B**) Assessment of the association between performance and age (both groups) and CGG repeat length (in the male fXPCs); no significant associations were seen.

One male HC did not complete the task, and responses from one male fXPC were identified as outliers, so they were removed from the analyses. In the remaining participants, the number of excluded trials (mean ± SD) was 3.24 ± 2.18 for HCs and 2.18 ± 2.04 for fXPCs, which did not differ significantly (t = 1.61, P = 0.12). There was no difference in error rates between the two groups (F_(1,40)_ = 0.0003, P = 0.99). The mean error rate ranged from 0% ± 0% for one item (no errors were committed) to 11.89% ± 13.30% for eight items for HCs and from 1.39% ± 3.93% to 13.33% ± 13.48%, respectively, for male fXPCs. Using a repeated-measures ANOVA, we found that there was a significant difference in reaction times between the two groups (F_(1,40)_ = 5.45, P = 0.02; see Figure 3A). Reaction times increased as the number of items increased (F_(7,280)_ = 257.68, P<0.001), and did not differ between the groups (F_(7,280)_ = 0.81, P = 0.60). This indicates that reaction times slowed as the number of items to enumerate increased, and both groups slowed at the same rate. A separate analysis did not correct for simple reaction time, and found that the main effect of group was no longer significant (P>0.05), whereas the main effect of numerosity remained significant, and the interaction between group and numerosity remained insignificant (results not shown). These contrasting results highlight that analyses accounting for simple reaction time differences are more sensitive to detecting group effects.

Analyses to identify the subitizing range from the counting range were performed. For the HCs, a significant quadratic trend emerged with one to four items, indicating a subitizing range from one to three items (F_(1,24)_ = 9.8, P = 0.004). Likewise, for the male fXPCs, a significant quadratic trend emerged with one to four items, indicating a subitizing range from one to three items (F_(1,16)_ = 10.06, P = 0.006). Because quadratic trends were identified over the range of one to four items for both groups, the same subitizing range (one to three items) and counting range (five to eight items) was used for the two groups. Because reaction time for four items was at a transition point between subitizing and counting, it was not included in any slope calculations. To summarize performance in the subitizing range, slopes for the linear fit lines through the points at one to three items were estimated for each participant. Similarly, for the counting range, slopes for the linear fit lines through the points at five to eight items were estimated for each participant.

To parse basic psychomotor speed from cognitive load, the median reaction time for each numerosity was divided by the median reaction time from the simple reaction time task, thus the slope is expressed in terms of an arbitrary unit. The mean subitizing range slope was 0.16 ± 0.08 AU/item for HCs and 0.09 ± 0.12 AU/item for male fXPCs. The mean counting range slope was 0.70 ± 0.44 AU/item for HCs and 0.84 ± 0.46 AU/item for male fXPCs. Slopes were different between the groups for the subitizing range (t = −2.34, P = 0.02) but not for the counting range (t = 1.03, P = 0.31). We also calculated the slopes using raw median reaction times to confirm that the subitizing range resulted in slopes of less than or equal to 100 ms/item and the counting range slope was greater than or equal to 250 ms/item. The mean subitizing range slope was 66.13 ± 34.07 ms/item for HCs and 36.09 ± 43.26 ms/item for male fXPCs (t = −2.51, P = 0.02). The mean counting range slope was 283.26 ± 165.34 ms/item for HCs and 325.65 ± 171.17 ms/item for male fXPCs (t = 0.80, P = 0.43). These data match the existing literature on expected subitizing and counting range slopes.

Further investigation into the slopes for the subitizing and counting range within each group assessed the association between these measures and age (both groups) and CGG repeat length (in the male fXPCs); there were no significant associations (Figure 3B). The correlation matrix is presented in Table 2.

## Discussion

The goal of this study was to determine whether young adult male fXPCs asymptomatic for FXTAS exhibit impairments in quantitatively relevant visuospatial processing, and we investigated this using two tasks: a quantitative magnitude comparison task and basic numerical enumeration task. We have previously shown that young adult female fXPCs show CGG-modulated and age-modulated spatiotemporal impairments, but have enhanced psychomotor speed, even though they have a second, unaffected FMR1 allele that is expressed randomly in 50% of their cells [14,40,41]. Because there is a spectrum of FMR1 involvement based on FMR1 dosage, in terms of CGG repeats and gender, we hypothesized that male fXPCs would be impaired relative to HCs and would be more cognitively affected than female fXPCs.

We found that male fXPCs and HCs had similar simple manual and oral psychomotor reaction times. This contrasts with our previous finding that female fXPCs produce enhanced psychomotor speed on the same tasks [14]. We also found that, even after controlling for psychomotor speed, male fXPCs were slower at judging relative magnitude and at enumerating items on a screen. This also contrasts with our previous findings that female fXPCs performed similarly to HCs [40,41]. Thus, our results support our hypothesis that male fXPCs are more cognitively affected than female fXPCs of a similar age and CGG repeat length range. We suggest that the slower reaction times in these cognitively demanding visuospatial tasks may be due to impairments in spatial and temporal processing, similar to the more pronounced attentional impairments reported in young individuals with FXS during visual search tasks [55,56]. Our laboratory has previously published data describing weaknesses across distinct neurogenetic disorders [52] and noted that although the genetic etiology of these disorders is different, the shared weaknesses in spatial and temporal processing domains present as a cascade of effects that leads to mathematical and numerical learning difficulties. These common phenotypes may be partially due to overlapping molecular mechanisms, as indicated by the finding that FMR1 mRNA with expanded CGG repeat length interacts with the DiGeorge syndrome critical region 8 (DGCR8) protein, which is haploinsufficient in chromosome 22q11.2 deletion syndrome [57]. Similarly, an underlying mechanism may explain why individuals with the FMR1 full mutation and those with the premutation, although they are thought to be affected via different mechanisms (FMRP deficiency and RNA toxicity, respectively), both exhibit visuospatial processing impairments.

### Relationship between CGG, age, and cognitive performance

To determine the effect of FMR1 dosage on performance, we assessed the association between behavioral measures and age (both groups) and CGG repeat length (in fXPCs). We found no associations between performance measures and age or CGG repeat length. This contrasts with our finding in female fXPCs in the same age range, in whom both age and CGG repeat length were associated with the intercept in the magnitude comparison task and with the counting range slope in the enumeration task [40,41]. However, owing to differences in behavioral performance, intercept ranges were not the same for males (10 to 30 mm) as for females (10 to 20 mm) in the magnitude comparison task, which may partially explain these different findings in males.

Because CGG repeat length is non-linearly related to FMR1 mRNA and FMRP levels, which are more direct measures of molecular function, it may be the case that CGG repeat length is non-linearly related to cognitive function. Indeed, several studies in female fXPCs have found evidence of a curvilinear relationship between CGG repeat length and major depressive disorder [58], sensitivity to life stress [59], age at menopause [60], and reproductive aging [61,62]. However, the CGG repeat range in our sample (55–118 repeats) was not sufficiently large to detect such a relationship.

It is possible that the lower CGG repeat range in the current study represents a subset of the fXPC phenotype (that is, slightly higher levels of FMR1 mRNA and normal FMRP), as fXPCs with more than 100 repeats have more pronounced levels of FMR1 mRNA elevation [63]. However, it is unknown how male fXPCs with higher CGG repeats (120 to 200; that is, those with higher levels of FMR1 mRNA and potentially reduced FMRP) would perform on these tasks. A recent study found that in adult male fXPCs in the upper premutation range (>100 CGG repeats), increasing age is associated with poorer performance on executive function tasks of inhibition and executive working memory, whereas men in the lower premutation range (<100 CGG repeats) were relatively unaffected [20]. Another study found that adult male fXPCs have intact perception as assessed by line orientation judgment and face gender identification tasks, but have impaired visuospatial performance as assessed by mental rotation and visuospatial working memory tasks [25]. Only visuospatial memory performance correlated with age, and this was only in fXPCs in the upper premutation range (>100 CGG repeats). These studies, which, like the present study examined only males asymptomatic for FXTAS, suggest that men in the upper premutation range may exhibit even more pronounced impairments in magnitude comparison and enumeration than men in the lower premutation range.

The data from the present study suggest that adult male fXPCs in the lower premutation range (our sample) exhibit cognitive slowing. Meanwhile, adult male fXPCs in the upper premutation range (other samples) seem to exhibit both accuracy decrements [20,25] and cognitive slowing that is additionally modulated by age. This possibility emphasizes the importance of examining both reaction time and accuracy as concurrent measures of performance. Whereas prior studies reported an age effect on performance in male fXPCs, we did not see such an age effect, possibly because our sample consisted of a younger and less variable age range (mean 30.72 ± 6.51 compared with 45 ± 14 years) [20,25].

### Relationship between cognitive performance and endophenotype in fXPCs

Our results support previous research indicating that fXPCs are impaired on tasks requiring processing of visual spatial and temporal information (spatiotemporal processing). For example, adult fXPCs have impairments on tasks that rely on the M pathway, but not on tasks that rely on the P pathway [43-45]. A mouse model of fXPC shows impaired memory for spatial locations [46], temporal order [47], and temporal order of spatial locations [48]. Whereas younger fXPC mice were impaired in detecting a change in distance between two objects, but not in detecting a transposition of objects, older fXPC mice were impaired in both tasks. This suggests that hippocampal-dependent impairments in spatial processing (for example, detecting a change in distance) may precede parietal cortex-dependent impairments (for example, detecting transposition) [46].

Because representations of space and time provide the foundation for an understanding of numbers [52], impaired spatiotemporal processing may underlie the observed impairments in arithmetic performance in fXPCs [39]. This is particularly notable because functional brain activation during arithmetic processing is related to FMRP expression in females with FXS, suggesting that the endophenotype of spatiotemporal processing ability in fXPCs may be sensitive to the molecular FMR1 phenotype. Finally, positron emission tomography imaging of female fXPCs has shown hypometabolism of the right parietal, temporal, and occipital associations areas [42], areas that subserve spatiotemporal processing. This suggests that spatiotemporal impairments may be due to abnormal functioning of these brain regions, even independent of task demand. Thus, our results are consistent with previous behavioral and neuroimaging findings in fXPCs across species.

## Conclusions

The results of the current study support previous findings of impaired performance in visuospatial tasks in male fXPCs [3,25,45]. It adds to a growing body of literature characterizing the phenotypic spectrum produced by FMR1 dosage modulations. Although adult male fXPCs did not differ from HCs in psychomotor speed, they were slower in judging relative magnitude and in enumerating items on a screen. This study may at least partially explain the lack of impairment reported in some studies that did not control for the effect of psychomotor speed in cognitive tasks. Further studies examining a larger range of CGG repeats and age may help identify whether distinct phenotypic subtypes of the premutation exist, which may aid in the early detection and prevention of neurodegenerative disease.

## Abbreviations

DGCR8: DiGeorge syndrome critical region 8; FMR1: fragile X mental retardation 1; FMRP: fragile X mental retardation protein; FSIQ: Full-scale IQ; fXPC: fragile X premutation carrier; FXS: fragile X syndrome; FXTAS: fragile X-associated tremor/ataxia syndrome; HC: Healthy control.

## Competing interests

The authors declare that they have no competing interests.

## Authors’ contributions

LMW performed the data interpretation and manuscript preparation; NJG performed data analysis, interpretation, and manuscript preparation; YM performed data collection and data interpretation; FT performed the molecular genetics analysis; DH provided advice regarding the data analysis; SMR participated in the design of the study, and supported the data interpretation and manuscript preparation; TJS conceived of the study, participated in design and coordination, and supported data interpretation and manuscript preparation. All authors read and approved the final manuscript.
